# *PIMREG* is a prognostic biomarker involved in immune microenvironment of clear cell renal cell carcinoma and associated with the transition from G1 phase to S phase

**DOI:** 10.3389/fonc.2023.1035321

**Published:** 2023-01-26

**Authors:** Huibao Yao, Feifei Lyu, Jian Ma, Fengze Sun, Gonglin Tang, Jitao Wu, Zhongbao Zhou

**Affiliations:** ^1^ Department of Urology, The Affiliated Yantai Yuhuangding Hospital of Qingdao University, Yantai, Shandong, China; ^2^ Department of Traditional Chinese Medicine, The Sixth Medical Center of PLA General Hospital, Beijing, China; ^3^ Department of Urology, Beijing TianTan Hospital, Capital Medical University, Beijing, China

**Keywords:** *PIMREG*, clear cell renal cell carcinoma, immune infiltration, drug sensitivity, prognosis, biomarker

## Abstract

**Background:**

Clear cell renal cell carcinoma (ccRCC) is one of the most common tumors in the world and affects human health seriously. *PIMREG* is a mitotic regulator which is essential to the metaphase-to-anaphase transition in cell cycle. Although *PIMREG* plays a crucial role in the malignant progression of tumors, there are few reports on its role in ccRCC.

**Methods:**

The transcriptional expression profile and clinical data of *PIMREG* were downloaded from TCGA database and verified by qRT-PCR. Kaplan-Meier plotter was used to analyze the effect of *PIMREG* on overall survival (OS), disease specific survival (DSS) and progression-free interval (PFI) of patients with ccRCC. Univariable and multivariable Cox regression analysis were used to determine the independent prognostic factors of ccRCC. The effects of *PIMREG* on cell migration and invasion were detected by wound healing assay and transwell invasion assay, and CCK-8 assay, colony formation assay and cell cycle assay were used to detect the effect of *PIMREG* on cell proliferation. In addition, the changes in cell cycle related proteins were detected by western blot.

**Results:**

*PIMREG* was highly expressed in human ccRCC and was positively correlated with pathologic stage, TNM stage and histologic grade. In addition, patients with high expression of *PIMREG* had a poor prognosis. Univariable and multivariable Cox regression analysis identified that *PIMREG* was an independent prognostic factor of ccRCC. Additionally, *PIMREG* was also closely related to immune cell infiltration. Experiments *in vitro* identified that the knockdown of *PIMREG* could significantly inhibit the proliferation, migration and invasion abilities of ccRCC. The expression of cyclin D1, CDK4 and CDK6 was also significantly reduced after *PIMREG* knockdown.

**Conclusions:**

*PIMREG* plays a vital role in the development of ccRCC and may become a potential therapeutic target in the future.

## 1 Introduction

Renal cell carcinoma (RCC) is one of the most common malignant tumors in the urinary system, which has a serious impact on people’s health (1). RCC, also known as renal adenocarcinoma, originates from renal tubular epithelial cells. The incidence rate of RCC in adult malignant tumors is 2% ~ 3% with the increasing trend year by year (2). The pathological types of RCC include clear cell carcinoma, papillary cell carcinoma and chromophobe cell carcinoma, among which clear cell renal cell carcinoma (ccRCC) accounts for 70% ~ 80% (3). Clear cell renal cell carcinoma is not sensitive to chemotherapy or radiotherapy, and surgery is the most effective treatment (4). However, nearly 30% of patients with ccRCC experience recurrence or metastasis after operation (5). Therefore, we must explore the molecular mechanism of ccRCC progression to provide new strategies for the diagnosis and treatment of ccRCC.

PICALM Interacting Mitotic Regulator (*PIMREG*), which is also known as FAM64A and CATS, is located at chromosome 17 band p13.2 (6). It was first reported in 2006 that the expression of *PIMREG* could significantly increase the nuclear localization of clathrin assembly lymphoid myeloid (CALM) and leukemia fusion protein CALM/AF10 (7). *PIMREG* has been proved to control the transition of cell mitosis from metaphase to anaphase and regarded as a marker of proliferation (8). Recent studies have found that the upregulation of *PIMREG* expression is related to the poor prognosis of breast cancer, neuroblastoma, cholangiocarcinoma, osteosarcoma, glioma, prostate cancer and other cancers (9–14). Although Wei et al. and Zhu et al. reported that *PIMREG* was related to overall survival (OS) and recurrence-free survival (RFS) of ccRCC and could be used as a biomarker of prognosis, we further verified the biological function of *PIMREG* in ccRCC through *in vitro* experiments and analyzed the relationship between *PIMREG* and immune infiltration and drug resistance (15, 16).

In this study, we analyzed a ccRCC dataset from the TCGA database and found that *PIMREG* was highly expressed in ccRCC and its expression was positively correlated with pathological stage and grade and poor prognosis of patients. In addition, silencing *PIMREG* inhibited the proliferation, migration and invasion of ccRCC *in vitro*. The above results suggested that *PIMREG* is a marker of poor prognosis of ccRCC and may become a potential diagnostic and therapeutic target of ccRCC.

## 2 Materials and methods

### 2.1 Data collection and process

The Cancer Genome Atlas (TCGA) database (https://portal.gdc.cancer.gov/) is a large public database containing genome and transcriptome data of 33 cancers. We downloaded gene expression and clinical information data of all 33 cancer types from TCGA for follow-up studies. To examine the differential expression of *PIMREG* in various cancers in the TCGA database, we normalized the mRNA expression profile using the “limma” package in R (version 4.0.4). We also used TIMER2 (http://timer.cistrome.org/) and The Human Protein Atlas (https://www.proteinatlas.org/) to investigate the difference of *PIMREG* expression between tumor and normal tissues.

### 2.2 Univariable, multivariable Cox regression analysis and logistic regression analysis

We plotted a Kaplan-Meier survival curve to determine the effect of *PIMREG* expression on the prognosis of patients with ccRCC. In addition, univariable and multivariable Cox regression analyses were performed to evaluate whether *PIMREG* and other clinical features (such as age, gender, T stage, N stage, M stage, pathological stage, and histologic grade) can be used as independent related factors affecting the prognosis of patients with ccRCC. Logistic regression analysis was conducted for all variables to calculate odds ratio (OR) and 95% confidence interval (CI), and further determine the risk factors for ccRCC.

### 2.3 Establishment of the nomogram

We used the “rms” package in R (version 4.0.4) to construct a nomogram based on independent risk factors screened to predict the OS of ccRCC patients at 1, 3 and 5 years. In addition, we constructed calibration curves to estimate the accuracy of the nomogram.

### 2.4 Protein-protein interaction (PPI) network and functional annotation analysis

String (https://cn.string-db.org/) was used to search and draw a PPI network of genes interacting with *PIMREG*. At the same time, the “clusterprofiler” package in R (version 4.0.4) was used for the enrichment analysis of Gene Ontology (GO) and Kyoto Encyclopedia of Genes and Genomes (KEGG). GO is divided into three parts in detail: molecular function (MF), biological process (BP), and cellular component (CC). All the analyses were visualized through the “ggplot2” package in R (version 4.0.4).

### 2.5 Analysis of immune cells infiltration and the immune microenvironment

CIBERSORT (https://cibersort.stanford.edu/) is a free database that can query the composition of immune cells in various tissues. TIMER2 can also be used to analyze immune cell infiltration. We examined the difference in the levels of immune cell infiltration between the groups with high and low PIMREG expression, as well as the relationship between PIMREG expression and 24 different types of immune cells. In addition, the correlation between PIMREG and different immune related genes was analyzed by the “corrplot” package in R (version 4.0.4).

### 2.6 Patient samples

We collected the ccRCC tissues and matched normal renal tissues from 32 patients who underwent radical or partial nephrectomy in the urology department of Qingdao University Affiliated Yantai Yuhuangding Hospital since 2020 to 2022. The samples collected during the operation were quickly placed in liquid nitrogen, and then moved to -80°C refrigerator for storage for subsequent use. All participants signed the informed consent form, and the project was authorized by the ethics committee of Qingdao University Affiliated Yantai Yuhuangding Hospital.

### 2.7 Cell lines and cell culture

The human normal renal proximal convoluted tubular cell line (HK-2) and ccRCC cell lines (786-O, 769-P, ACHN, Caki-2, and A498) were purchased from the Cell bank of Chinese Academy of Sciences. HK-2 cells were cultured with DMEM (BI, Israel) and other cells were cultured with RPMI 1640 (BI, Israel). All the media were added with 10% fetal bovine serum (FBS) and 1% penicillin and streptomycin. All the cells were cultured in a humidified incubator at 37°C and 5% carbon dioxide.

### 2.8 Transfection

Small interfering *PIMREG* RNA (siR-*PIMREG*) and its corresponding negative control siRNAs (NC) were synthesized by General Biol Company (China). NC and siR-*PIMREG* were transfected into ccRCC cell lines using Lipofectamine 3000 (Invitrogen, USA). All the primers and oligonucleotide sequences were listed in **Table 1**.

**Table 1:** Primer sequences used in this research.

Name	Sequence
GAPDH(human)-F	GTCTCCTCTGACTTCAACAGCG
GAPDH(human)-R	ACCACCCTGTTGCTGTAGCCAA
PIMREG(human)-F	CGCTCAGCTAAGAGTGCTTTGG
PIMREG(human)-R	TGCCCTTCTGTGCTCCTCTCTT
NC(human)-F	UUCUCCGAACGUGUCACGUTT
NC(human)-R	ACGUGACACGUUCGGAGAATT
siR-PIMREG(human)-F	GGCUCACAUGCCCACCCAUTT
siR-PIMREG(human)-R	AUGGGUGGGCAUGUGAGCCTT

### 2.9 RNA extraction, reverse transcription and quantitative real−time PCR (qRT-PCR)

We extracted total RNA from freshly frozen tissues or ccRCC cell lines using SteadyPure Quick RNA Extraction Kit (Accurate Biology, China) and then conducted reverse transcription using Evo M-MLV RT Mix Kit (Accurate Biology, China). Next, we carried out qRT-PCR by using SYBR^®^ Green Premix Pro Taq HS qPCR Kit and Rox Reference Dye (Accurate Biology, China). The housekeeping gene GAPDH was used as the internal reference, and the relative expression level of the target gene was calculated by the 2^−ΔΔCT^ calculation method. Three multiple holes were set for each experiment, and the primer sequences used were listed in **Table 1**.

### 2.10 Western blot

The cells were lysed in RIPA lysis buffer (Servicebio, China) containing 1% protease inhibitor for 30 minutes, and the protein supernatant was obtained after centrifugation. The supernatant was then mixed with 5× protein loading buffer (Coolaber, China) and heated at 100°C for 10 minutes in preparation for sample loading. Electrophoresis was carried out by SDS-PAGE and the proteins were then moved to NC membrane. The membranes were blocked with TBST buffer containing 5% skimmed milk powder for 2 hours and then placed in a 4°C shaking table overnight with various primary antibodies. The next day, the membrane was incubated with secondary antibody at room temperature for one hour. A Clinx Gel Documentation and Analysis instrument was used to observe the protein bands, and ImageJ was used to analyze the gray value of the bands. The antibodies used were as follows: anti-cyclin D1 (Cell Signaling Technology, USA, 1:1000), anti-CDK4 (Cell Signaling Technology, USA, 1:1000), anti-CDK6 (Cell Signaling Technology, USA, 1:1000), anti-vinculin (Abmart, China, 1:1000), anti-β-tubulin (Cell Signaling Technology, USA, 1:1000), goat anti-rabbit secondary antibody (Cell Signaling Technology, USA, 1:5000), and goat anti-mouse secondary antibody (Cell Signaling Technology, USA, 1:5000).

### 2.11 Cell counting kit−8 (CCK−8) assay

786-O and ACHN cells transfected with NC and siR-*PIMREG* were plated in 96-well plates at a density of 2×10^3^ cells per well. CCK-8 reagent was added at 0, 24, 48, 72 and 96h respectively. After incubating at 37°C for 2 hours, the absorbance of cells was measured at 450 nm wavelength with a spectrophotometer.

### 2.12 Colony formation assay

786-O and ACHN cells transfected with NC and siR-*PIMREG* were plated in 6-well plates at a density of 1000 cells per well. After 14 days of culture, the cells were fixed with 4% paraformaldehyde for 15 minutes. Then Giemsa staining (Solarbio, China) was used for 30 minutes, and the colonies were photographed and counted.

### 2.13 Cell cycle assay

786-O and ACHN cells transfected with NC and siR-*PIMREG* were collected and rinsed twice with PBS after centrifugation. Then 5ml of 75% cold ethanol was added dropwise to the cells and the cells were fixed at -20°C for at least 2 hours. The fixed cells were re-suspended with 0.5ml PI/RNase staining buffer and incubated for 15 minutes. The cell cycle was detected by flow cytometry and analyzed by ModfitLT software.

### 2.14 Wound healing assay

The cells were inoculated into 6-well plates at the ratio of 2×10^6^ per well and cultured for 12 hours until they adhered to the wall. A 1000μl sterile straw tip was used to draw a line in the horizontal and vertical directions. After cleaning the orifice plate with phosphate buffer, the images were collected immediately (0h point) using microscope. Then, 2ml RPMI 1640 complete medium was added to each well for incubation and images were taken again at the same position after 12h. The length of wound healing was used as an indicator of cell migration.

### 2.15 Transwell invasion assay

We used a transwell plate with an 8μm diameter polycarbonate film to evaluate the invasion ability of cells. Then, 70μl Matrigel matrix was applied on the upper layer of the membrane 2 hours in advance. The cells were resuspended with serum-free medium and planted on the Matrigel matrix (5×10^4^ cells per well), and the complete medium was used in the lower compartment. After incubation for 24-72 h, the cells were fixed with methanol, stained with 0.5% Giemsa stain (Solarbio, China) and then photographed and counted under the microscope.

### 2.16 Statistics analysis

Wilcoxon rank sum test and unpaired t test were used to compare the difference in the PIMREG expression between two groups. In the clinical pathological characteristics table, the difference between four-category ordinal variable (T stage, pathologic stage and histologic grade) and two-category variable (N stage, M stage, gender and age) were compared by Cochran-Armitage test and chi-square test respectively. All the experiments in this paper were repeated three times, and the final experimental data were expressed as mean ± standard deviation. Unpaired t-test and one-way ANOVA analysis were used to analyze the differences between experiment and control groups. Pearson’s test and Spearman’s test were used for correlation analysis. All the statistical analyses were performed using GraphPad Prism 8. P < 0.05 was considered statistically significant.

## 3 Results

### 3.1 *PIMREG* expression is up-regulated in ccRCC in the database

**Figure 1:**
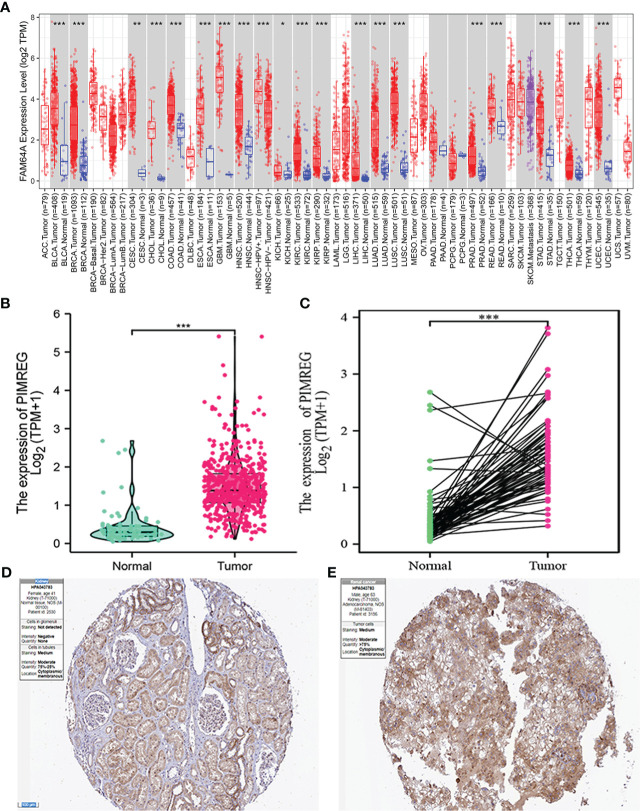
The mRNA and protein expression levels of *PIMREG* in ccRCC. **(A)** The mRNA expression levels of *PIMREG* in pan-cancer from TIMER2 database. **(B)** The mRNA expression levels of *PIMREG* in TCGA ccRCC database (T = 539, N = 72). **(C)** The *PIMREG* mRNA expression of pairwise ccRCC samples in TCGA dataset (T = 72, N = 72). **(D)** The protein expression levels of *PIMREG* in normal kidney tissues based on the Human Protein Atlas. **(E)** The protein expression levels of *PIMREG* in ccRCC tissues based on the Human Protein Atlas. *P < 0.05, **P < 0.01, ***P < 0.001; ccRCC: clear cell renal cell carcinoma.

We discovered that *PIMREG* displayed an elevated trend in 19 different tumor types by analyzing the TIMER2 database, including ccRCC (**Figure 1A**). Subsequently, we analyzed the expression level of *PIMREG* in 539 renal cancer tissues and 72 normal renal tissues in TCGA database. The results identified that the expression of *PIMREG* in ccRCC was significantly higher than that in adjacent normal tissues (P <0.001, **Figure 1B**) and paired data also supported this finding (P <0.001, **Figure 1C**). In addition, immunohistochemistry staining in the Human Protein Atlas indicated that *PIMREG* protein was similarly upregulated in ccRCC (**Figures 1D, E**). These results identified that the *PIMREG* expression was up-regulated at both the mRNA and protein levels in ccRCC.

### 3.2 Relationship between *PIMREG* expression and clinical pathological features

**Figure 2:**
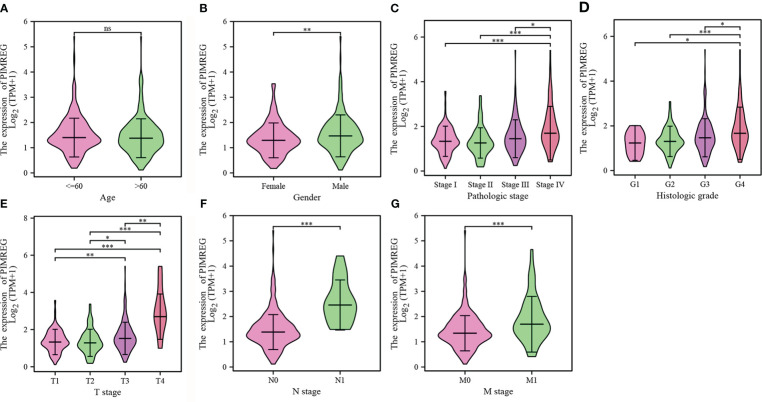
Associations between the *PIMREG* expression levels and clinical characteristics. **(A)** age; **(B)** gender; **(C)** pathologic stage; **(D)** histologic grade; **(E)** T stage; **(F)** N stage; **(G)** M stage. *P < 0.05, **P < 0.01, ***P < 0.001, ns: no significance.

Five hundred and thirty-nine ccRCC tissues in TCGA database were divided into a high expression group and a low expression group according to the median value of *PIMREG* expression. **Table 2** displays the specific baseline clinical and pathological characteristics of the patients. As shown in **Figures 2A–G**, there was a significant correlation between the expression of *PIMREG* and gender (P < 0.01), pathological stage (P < 0.05), histologic grade (P < 0.05), T stage (P < 0.05), N stage (P < 0.001), and M stage (P < 0.001), and the expression of *PIMREG* gradually increased with increasing pathological stage, histological grade, and TNM stage. Age and the expression of *PIMREG*, however, did not significantly correlate. In general, patients with high *PIMREG* expression had a higher degree of tumor malignancy, suggesting that *PIMREG* may be a potential factor for poor prognosis of ccRCC.

**Table 2:** The detailed baseline clinical pathological characteristics of the patients.

Characteristic	Low expression of PIMREG n=269	High expression of PIMREG n=270	P value
T stage, n (%)			
T1	152 (28.2%)	126 (23.4%)	< 0.001^*^
T2	42 (7.8%)	29 (5.4%)	
T3	74 (13.7%)	105 (19.5%)	
T4	1 (0.2%)	10 (1.9%)	
N stage, n (%)			
N0	120 (46.7%)	121 (47.1%)	**< 0.001^#^**
N1	0 (0%)	16 (6.2%)	
M stage, n (%)			
M0	229 (45.3%)	199 (39.3%)	**< 0.001^#^**
M1	21 (4.2%)	57 (11.3%)	
Pathologic stage, n (%)			
Stage I	149 (27.8%)	123 (22.9%)	< 0.001^*^
Stage II	38 (7.1%)	21 (3.9%)	
Stage III	59 (11%)	64 (11.9%)	
Stage IV	23 (4.3%)	59 (11%)	
Gender, n (%)			
Female	109 (20.2%)	77 (14.3%)	**0.016^#^**
Male	160 (29.7%)	193 (35.8%)	
Age, n (%)			
<=60	132 (24.5%)	137 (25.4%)	0.763^#^
>60	137 (25.4%)	133 (24.7%)	
Histologic grade, n (%)			
G1	8 (1.5%)	6 (1.1%)	< 0.001^*^
G2	139 (26.2%)	96 (18.1%)	
G3	95 (17.9%)	112 (21.1%)	
G4	22 (4.1%)	53 (10%)	

#The data were analyzed using chi-square test.

*The data were analyzed using Cochran-Armitage test. Bold font is used to indicate that the difference is statistically significant.

### 3.3 High expression of *PIMREG* indicates worse prognosis

**Figure 3:**
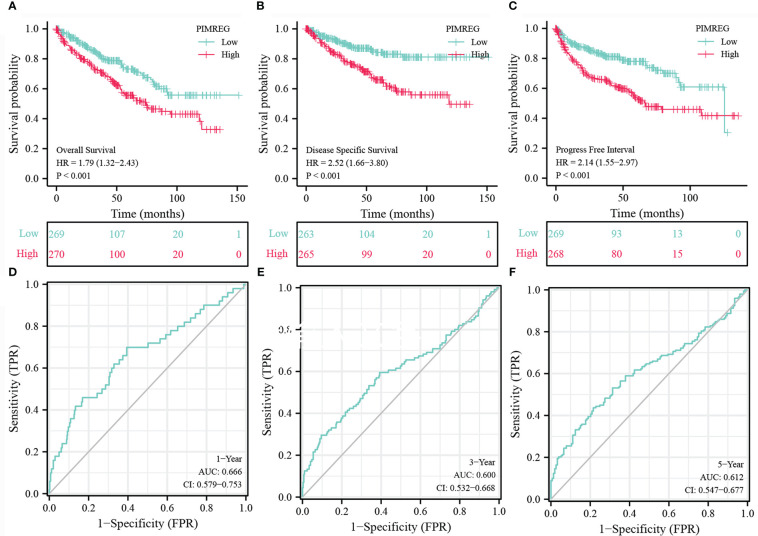
Analysis about Kaplan-Meier and ROC curves for *PIMREG*. **(A-C)** Kaplan-Meier survival curves indicated that ccRCC patients with high *PIMREG* mRNA expression had a shorter OS, DSS and DFI than the low *PIMREG* mRNA expression group. **(D-F)** ROC curve predicted the accuracy of OS in 1-, 3-, 5-years. ccRCC, clear cell renal cell carcinoma; OS, overall survival; DSS, disease specific survival; PFI, progression-free interval.

**Figure 4:**
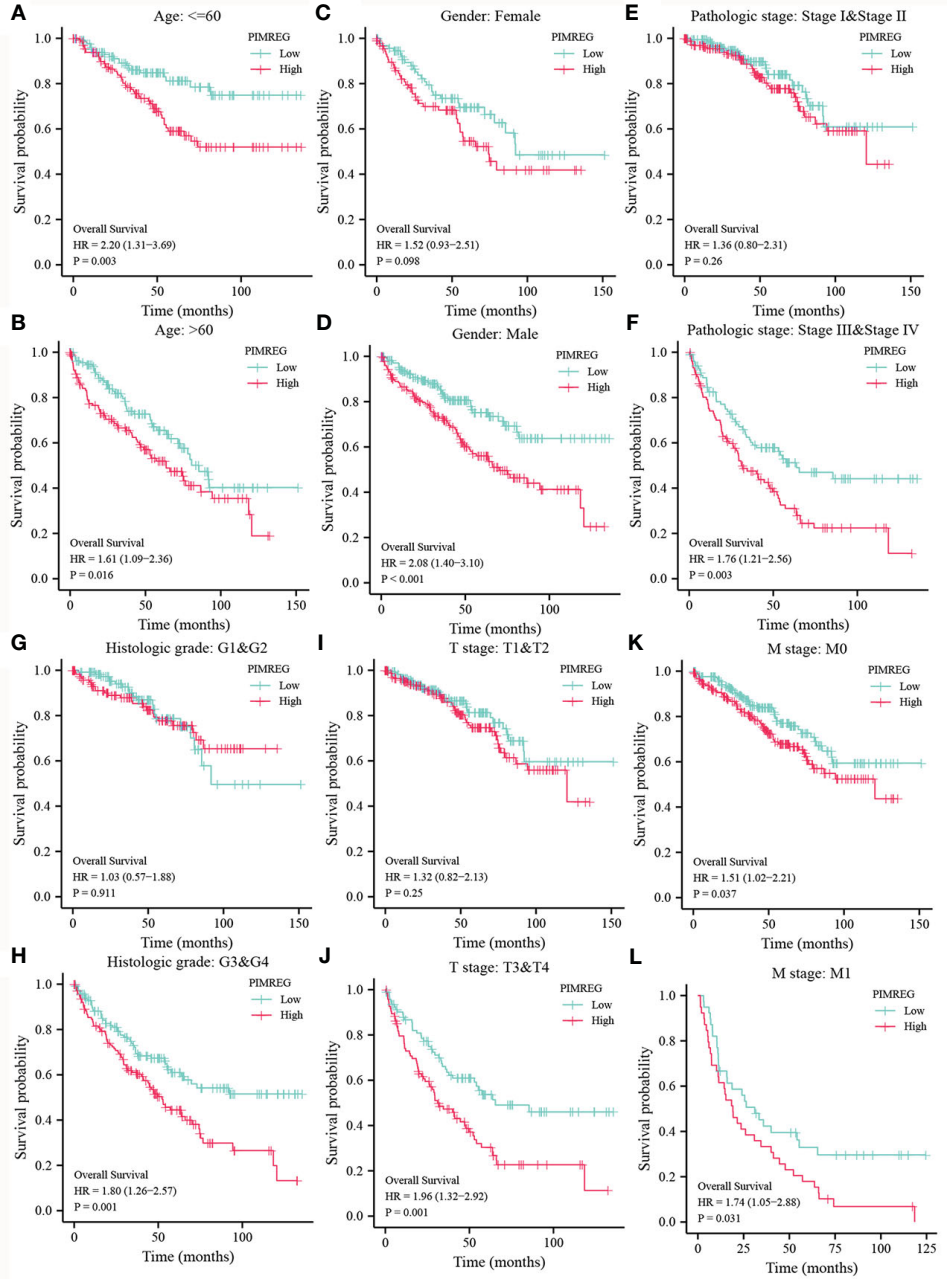
Subgroup analysis of OS according to different clinical characteristics. **(A, B)** age: > 60 or ≤ 60; **(C, D)** gender: female or male; **(E, F)** pathologic stage: stage I & stage II or stage III & stage IV; **(G, H)** histologic grade: G1&G2 or G3&G4; **(I, J)** T stage: T1&T2 or T3&T4; **(K, L)** M stage: M0 or M1.

We detect a relationship between *PIMREG* expression and OS, DSS (disease specific survival) and PFI (progression-free interval) in patients with ccRCC using Kaplan-Meier curves. **Figures 3A–C** indicated that compared with low *PIMREG* expression patients, the OS, DSS and PFI of ccRCC patients with high *PIMREG* expression were significantly shorter (OS: HR = 1.79(1.32-2.43), P < 0.001; DSS: HR=2.52(1.66-3.80), P < 0.001; PFI: HR=2.14(1.55-2.97), P < 0.001). The AUC values at 1 year, 3 years and 5 years were 0.666, 0.600 and 0.612 respectively according to the results of Receiver Operating Characteristic (ROC) curves, indicating that the prediction model was reliable (**Figures 3D–F**). In addition, we also performed subgroup analysis of OS according to age, gender, pathological stage, histologic grade, T stage and M stage. The results showed that the OS of patients with high *PIMREG* expression was worse than patients with low *PIMREG* expression in the subgroups of age ≤ 60, age > 60, male, stage III and IV, grade 3 and 4, T3 and T4, M0 and M1, while there was no difference in the subgroups of female, stage I and II, grade 1 and 2, T1 and T2 (**Figures 4A–L**).

### 3.4 *PIMREG* may be an independent prognostic factor for ccRCC

We next performed univariable and multivariable Cox regression analysis to identify independent factors that may be highly related to ccRCC OS from *PIMREG* and other clinical characteristics (age, gender, T stage, N stage, M stage, pathologic stage, histologic grade). Univariable Cox regression analysis showed that age (P < 0.001), T stage (P < 0.001), N stage (P < 0.001), M stage (P < 0.001), pathologic stage (P < 0.001), histologic grade (P < 0.001) and *PIMREG* expression (P < 0.001) were significantly associated with OS. Multivariable Cox regression analysis indicated that age (P = 0.006), M stage (P < 0.001) and *PIMREG* expression (P < 0.0001) were independent prognostic factors for patients with ccRCC (**Table 3**). Additionally, the results of logistic regression model indicated that male, higher pathological stage and histologic grade led to worse prognosis (**Table 4**). Based on the above results, elevated *PIMREG* expression may serve as a valuable biomarker for a poor prognosis in ccRCC.

**Table 3:** Univariable and multivariable Cox regression analysis in *PIMREG* expression and clinical pathological characteristics of patients.

Characteristics		Total(N)	Univariate analysis		Multivariate analysis	
			Hazard ratio (95% CI)	P value	Hazard ratio (95% CI)	P value
Age N=539	<=60	269	1.765 (1.298-2.398)	**<0.001**	1.847 (1.195-2.853)	**0.006**
	>60	270				
Gender N=539	Female	186	0.930 (0.682-1.268)	0.648	0.996 (0.639-1.553)	0.985
	Male	353				
T stage N=539	T1&T2	349	3.228 (2.382-4.374)	**<0.001**	1.427 (0.626-3.255)	0.398
	T3&T4	190				
N stage N=257	N0	241	3.453 (1.832-6.508)	**<0.001**	1.282 (0.630-2.610)	0.493
	N1	16				
M stage N=506	M0	428	4.389 (3.212-5.999)	**<0.001**	2.627 (1.557-4.432)	**<0.001**
	M1	78				
Pathologic stage N=536	Stage I &Stage II	331	3.946 (2.872-5.423)	**<0.001**	1.227 (0.484-3.111)	0.667
	Stage III &Stage IV	205				
Histologic grade N=531	G1&G2	249	2.702 (1.918-3.807)	**<0.001**	1.441 (0.860-2.413)	0.165
	G3&G4	282				
PIMREG		539	2.796 (2.197-3.557)	**<0.001**	2.014 (1.364-2.974)	**<0.001**

Bold font is used to indicate that the difference is statistically significant.

**Table 4:** Logistic regression analysis of prognosis and clinical pathological characteristics of patients.

Characteristics	Total(N)	Odds Ratio (OR)	P value
Age (>60 vs. <=60)	539	0.993 (0.708-1.392)	0.966
Gender (Male vs. Female)	539	1.955 (1.364-2.814)	**<0.001**
Pathologic stage (Stage III&Stage IV vs. Stage I&Stage II)	536	2.080 (1.461-2.974)	**<0.001**
Histologic grade (G3&G4 vs. G1&G2)	531	2.196 (1.554-3.115)	**<0.001**
T stage (T3&T4 vs. T1&T2)	539	2.198 (1.534-3.167)	**<0.001**
N stage (N1 vs. N0)	257	14.876 (2.944-271.116)	**0.009**
M stage (M1 vs. M0)	506	2.875 (1.717-4.964)	**<0.001**

Bold font is used to indicate that the difference is statistically significant.

### 3.5 Construction and verification of nomogram based on *PIMREG* expression

**Figure 5:**
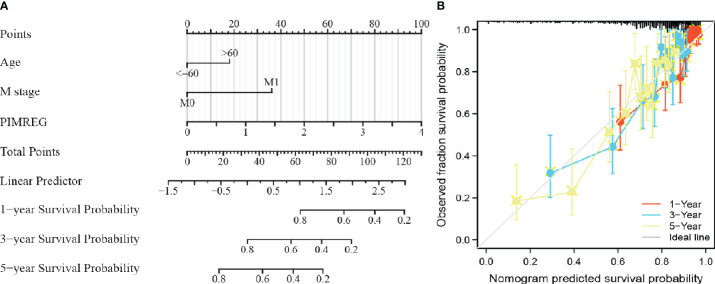
Establishment of a prognostic prediction nomogram and calibration plots about ccRCC. **(A)** Nomogram for predicting 1-, 3-, and 5-year OS probability for ccRCC patients. **(B)** Calibration plot of the nomogram for predicting the 1-, 3-, and 5-year OS probability. ccRCC: clear cell renal cell carcinoma; OS: overall survival.

To help clinicians better predict the prognosis of patients with ccRCC, we screened the clinical factors related to the prognosis based on multivariable Cox regression analysis and established a nomogram (age, M stage and *PIMREG*; **Figure 5A**). We defined a line segment in the nomogram we created to represent a score between 0 and 100. Each clinical factor’s line segment length and corresponding score illustrates its impact on the overall survival rate. Finally, the total score was obtained by adding the scores of each clinical factor, and the expected survival rate of patients at 1, 3 and 5 years was determined according to the total score. Additionally, the calibration curve indicated that there was a good agreement between the predicted and actual results (**Figure 5B**). In general, we successfully constructed a nomogram, a model that could accurately predict the prognosis of ccRCC patients.

### 3.6 Correlation genes and Functional annotation of *PIMREG*

**Figure 6:**
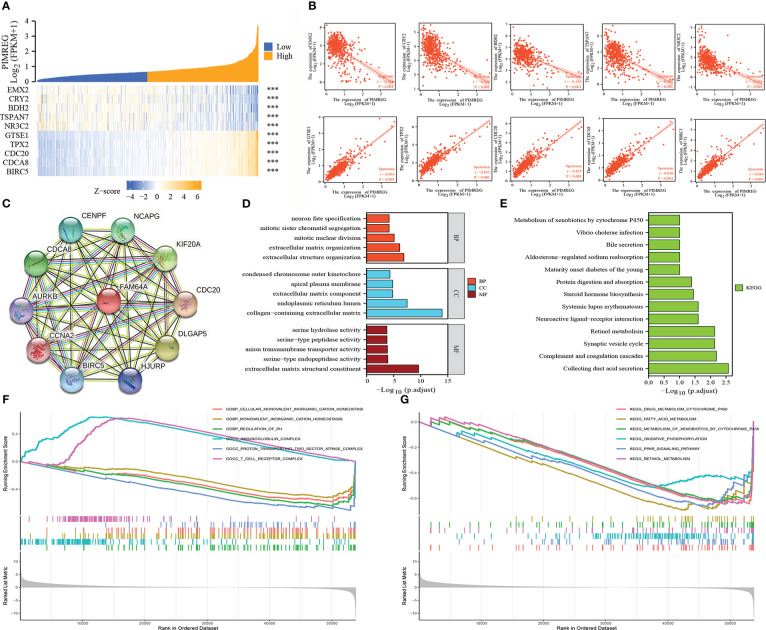
Related genes and functional annotation of *PIMREG*. **(A, B)** The first five genes that are positively or negatively correlated with the expression of *PIMREG*. **(C)** A network of *PIMREG* and its correlated genes. **(D, E)** GO and KEGG enrichment analyses about *PIMREG*. **(F, G)** GO term and KEGG pathway enrichment plots from GSEA analysis. GSEA, Gene Set Enrichment Analysis; GO, Gene Ontology; KEGG, Kyoto Encyclopedia of Genes and Genomes; ***P < 0.001.

We respectively selected the top five genes with the highest correlation from the positive or negative correlation genes with *PIMREG* and drew them into a heatmap. The results indicated that *PIMREG* was negatively correlated with EMX2, CRY2, BDH2, TSPAN7 and NR3C2 and positively correlated with GTSE1, TPX2, CDC20, CDCA8 and BIRC5 (**Figures 6A, B**). Based on the above results, we constructed the PPI network of *PIMREG* and its related genes using the STRING database (**Figure 6C**). We then annotated the function of *PIMREG* by GO and KEGG analyses. GO enrichment analysis suggested that *PIMREG* was related to the construction of the extracellular matrix, while *PIMREG* was believed to be associated to ligand-receptor binding and substance metabolism by KEGG enrichment analysis (**Figures 6D, E**). We also performed Gene Set Enrichment Analysis (GSEA) to further investigate the potential biological roles of *PIMREG*. The GSEA results showed that *PIMREG* was closely related to oxidative phosphorylation, fatty acid and retinol metabolism, and the formation of immunoglobulin complexes (**Figures 6F, G**).

### 3.7 Relationship between *PIMREG* and immune microenvironment

**Figure 7:**
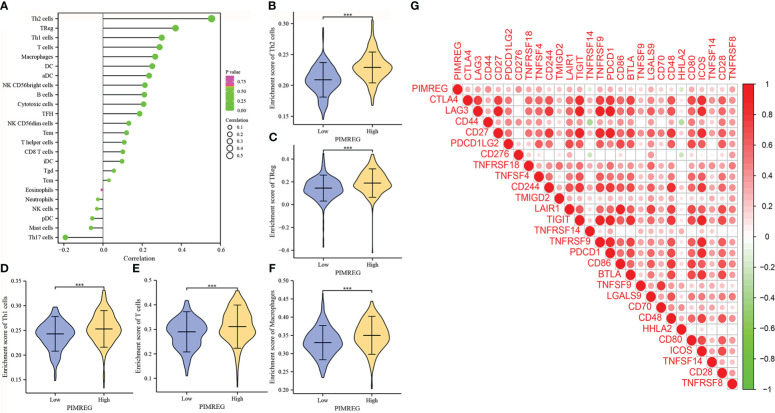
*PIMREG* is associated with immune cell infiltration and immune related genes. **(A)** Correlations of *PIMREG* expression levels with immune infiltration level. **(B-F)** *PIMREG* expression is positively related to enrichment scores of Th2 cells, TReg, Th1 cells, T cells and macrophages in ccRCC. **(G)** Corrplot graph shows the correlation between *PIMREG* and different immune related genes. ccRCC: clear cell renal cell carcinoma; ***P < 0.001.

According to our research, *PIMREG* was positively correlated with Th2 cells, Tregs, Th1 cells, T cells, macrophages, DCs and other immune cells, while it was negatively correlated with Th17 cells, mast cells, PDCs, NK cells and neutrophils (**Figure 7A**). The differences in the enrichment scores of different immune cells in the groups with high and low *PIMREG* expression were detailed in **Figures 7B–F**. In addition, the results of gene expression correlation analysis showed that *PIMREG* was significantly positively correlated with a variety of immune related genes including CD44, CD276, TNFSF4, and TNFRSF8 (**Figure 7G**). These results suggested that *PIMREG* may play an important role in the immune microenvironment in ccRCC.

### 3.8 Drug sensitivity analysis

**Figure 8:**
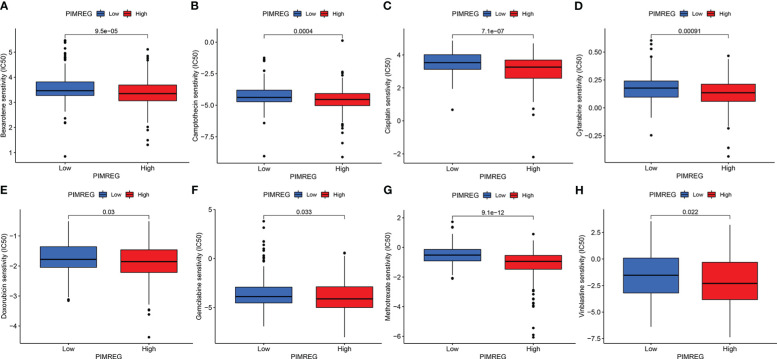
The sensitivity of patients in high *PIMREG* expression group and low expression group to different drugs. **(A)** bexarotene, **(B)** camptothecin, **(C)** cisplatin, **(D)** cytarabine, **(E)** doxorubicin, **(F)** gemcitabine, **(G)** methotrexate, **(H)** vinblastine.

We compared the sensitivity of different chemotherapeutic drugs between the high expression group and the low expression group of *PIMREG*, and the results indicated that the IC50 values of various drugs were all significantly different between the two groups. The IC50 values of bexarotene, camptothecin, cisplatin, cytarabine, doxorubicin, gemcitabine, methotrexate, and vinblastine in the high *PIMREG* expression group were significantly lower than those in the low *PIMREG* expression group (**Figure 8**). This suggested that the common chemotherapy drugs including cisplatin, cytarabine, gemcitabine may be effective in the treatment of *PIMREG*-overexpression ccRCC patients.

### 3.9 High-expression *PIMREG* in ccRCC tissues and cell lines

**Figure 9:**
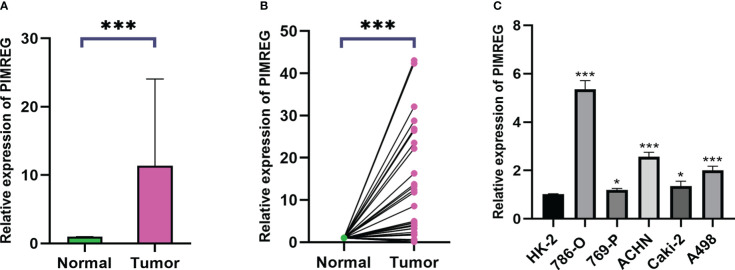
The mRNA expression levels of *PIMREG* in ccRCC tissues and cell lines. **(A, B)** The mRNA expression levels of *PIMREG* in ccRCC tissues (T = 32, N = 32). **(C)** The mRNA expression levels of *PIMREG* in ccRCC cell lines. ccRCC: clear cell renal cell carcinoma; *P < 0.05, ***P < 0.001.

We collected 32 pairs of tumor tissues and adjacent tissues from ccRCC patients for qRT-PCR verification. The baseline clinical pathological characteristics of the 32 patients were listed in **Supplementary Table S1**. The results identified that the mRNA expression level of *PIMREG* in tumor tissues was significantly higher than adjacent normal tissues (**Figures 9A, B**). We also confirmed it in renal cancer cell lines. The results demonstrated that compared with HK-2, the levels of *PIMREG* mRNA expression were higher in all renal cancer cell lines (**Figure 9C**: 786-O, P < 0.001; 769-P, P < 0.05; ACHN, P < 0.001; Caki-2, P < 0.05; A498, P < 0.001). We finally selected 786-O and ACHN cell lines with relatively higher *PIMREG* expression for further study.

### 3.10 *PIMREG* promotes ccRCC cells migration and invasion *in vitro*

**Figure 10:**
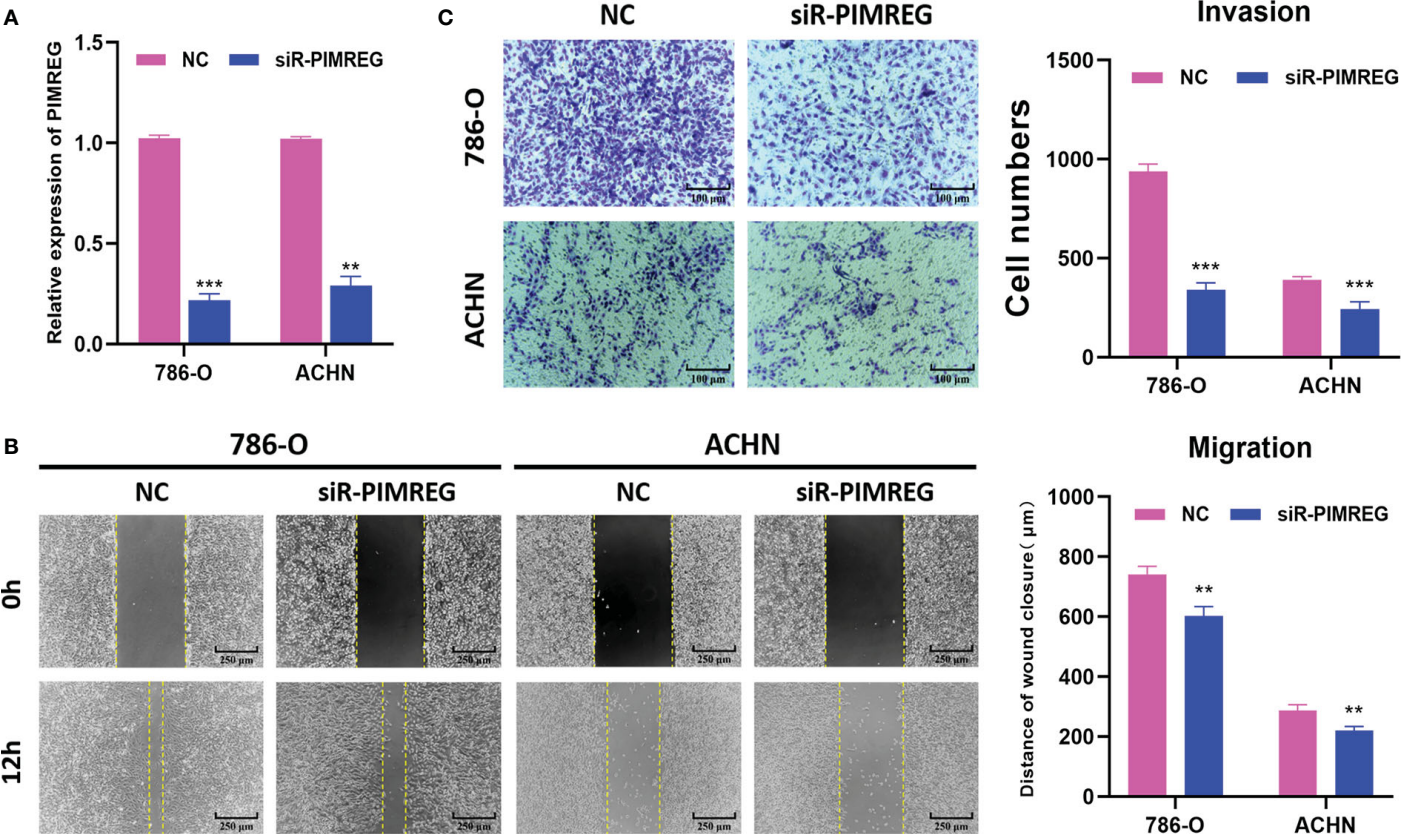
*PIMREG* promotes ccRCC cells migration and invasion *in vitro*. **(A)** The mRNA expression level of *PIMREG* was measured by qRT-PCR after cell transfection. **(B)** Wound healing assay indicated that knockdown of *PIMREG* reduced the migratory ability of ccRCC cells. **(C)** Transwell invasion assay indicated that knockdown of *PIMREG* reduced the invasive activity of ccRCC cells. ccRCC: clear cell renal cell carcinoma; **P < 0.01, ***P < 0.001.

To explore the biological function of *PIMREG* in ccRCC, we transfected siR-*PIMREG* and NC into 786-O and ACHN cell lines respectively. The knockdown efficiency was verified by qRT-PCR and the results showed that the expression of *PIMREG* was significantly reduced at the mRNA level (**Figure 10A**). Then, we detected the effect of *PIMREG* on the migration and invasion of ccRCC by wound healing experiment and transwell invasion experiment. Wound healing experiments indicated that the migration ability of 786-O and ACHN cells decreased significantly after *PIMREG* knockdown (**Figure 10B**). In addition, the results of transwell invasion experiments showed that the downregulation of *PIMREG* significantly inhibited the invasive ability of ccRCC (**Figure 10C**). These results identified that *PIMREG* could promote the migration and invasion of ccRCC *in vitro*.

### 3.11 *PIMREG* accelerated the ccRCC cells proliferation by promoting transition from G1 phase to S phase

**Figure 11:**
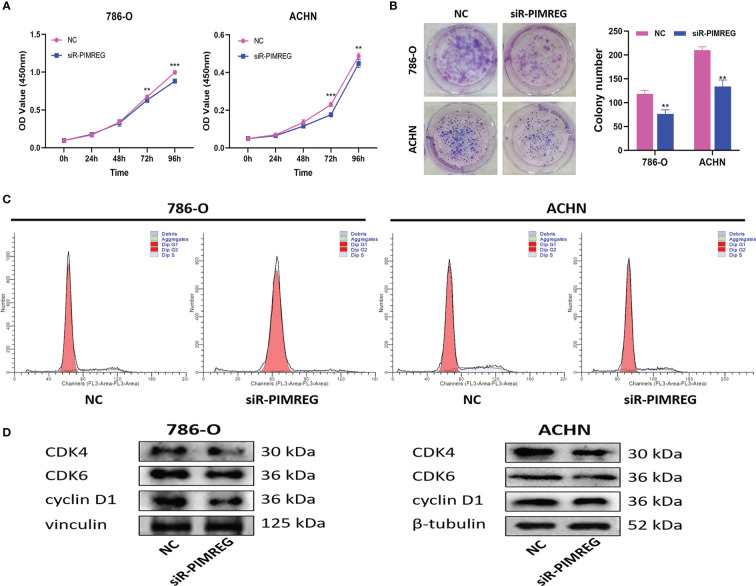
*PIMREG* promotes ccRCC cells proliferation *in vitro*. **(A)** CCK-8 assay indicated that knockdown of *PIMREG* reduced the proliferation ability of ccRCC cells. **(B)** Colony formation assay indicated that knockdown of *PIMREG* reduced the proliferation ability of ccRCC cells. **(C)** Flow cytometry indicated that knockdown of *PIMREG* inhibit the cells transition from G1 phase to S phase. **(D)** Western blot indicated that the expression levels of cell cycle related proteins decreased significantly after knockdown of *PIMREG*. ccRCC: clear cell renal cell carcinoma; **P < 0.01, ***P < 0.001.

Subsequently, we explored the effects of *PIMREG* on the proliferation and cell cycle of ccRCC through CCK-8 experiment, clone formation experiment and cell cycle experiment. The results of CCK-8 experiment and clone formation experiment showed that silencing *PIMREG* significantly weakened the proliferation (**Figure 11A**) and colony forming ability (**Figure 11B**) of 786-O and ACHN cells. According to the results of flow cytometry (**Figure 11C**), silencing *PIMREG* significantly increased the percentage of G1 phase cells (786-O: 83.39 ± 2.5% vs 91.01 ± 2.9%, P < 0.05; ACHN: 77.95 ± 2.8% vs 84.00 ± 2.4%, P < 0.05) and decreased the percentage of S phase cells (786-O: 16.15 ± 1.8% vs 8.99 ± 2.3%, P < 0.05; ACHN: 22.05 ± 1.6% vs 16.00 ± 1.7%, P < 0.05) in 786-O and ACHN cells. In addition, western blot also identified that the expression of cell cycle markers including cyclin D1, CDK4 and CDK6 was also decreased due to *PIMREG* silencing (**Figure 11D**). These results showed that *PIMREG* accelerated the process of cell cycle by facilitating the transition from G1 phase to S phase of cell cycle, and promoted the proliferation of ccRCC.

## 4 Discussion

Clear cell renal cell carcinoma is a common urological tumor, and its incidence rate has increased year by year. Because of insensitivity to radiotherapy and chemotherapy, radical nephrectomy has become an effective treatment for ccRCC. However, recurrence and metastasis after operation are still the main reasons for poor prognosis (17). Therefore, it is crucial to investigate the pathogenesis of ccRCC and find new therapeutic targets.

This study found that the expression of *PIMREG* in ccRCC tissue samples was significantly higher than that in adjacent normal tissues and was positively correlated with the high clinical stage and pathological grade of tumors through the analysis of TCGA database. Kaplan-Meier curve and Cox regression analysis showed that the increased expression of *PIMREG* was an important factor affecting the poor prognosis of patients with ccRCC. In addition, *PIMREG* played an important role in the infiltration of immune cells and the construction of extracellular matrix in ccRCC. *In vitro* experiments demonstrated that knockdown of *PIMREG* could significantly inhibit the proliferation, migration and invasion of ccRCC and slow down the procession of cell cycle. These results suggested that *PIMREG* may be used as a novel biomarker of ccRCC for diagnosis and treatment in the future.

Cell cycle related markers are considered to be the key drivers of malignant transformation of many types of tumors (18–20). *PIMREG* protein level is closely related to cell cycle and mitosis, which can be considered as a marker of cell proliferation and regulate this process (6). Numerous studies have shown that the expression of *PIMREG* is closely related to the progression of malignant tumors. Cyclin D1, one of the crucial cell cycle regulators, can drive cell transition from G1 phase to S phase and promote cell proliferation (21). Cyclin D1 is often overexpressed in several malignancies and is associated with the proliferation activity and poor prognosis (22). The activities of cyclin dependent kinases 4 and 6 (CDK4 and CDK6) are also essential to the cell cycle. They form a complex with cyclin D1 to jointly regulate tumor proliferation, and this complex is becoming a new target for tumor therapy (23, 24). Previous studies have reported that the knockdown of *PIMREG* can inhibit the proliferation of prostate cancer, glioma and breast cancer cells by prolonging G1 cell phase (9, 13, 14). In our study, the expression of cyclin D1 in ccRCC cells decreased significantly after silencing *PIMREG*. In addition, the percentage of G1 phase cells increased, while the percentage of S phase cells decreased significantly. This result shows that *PIMREG* can accelerate the progression of ccRCC by promoting the transition of cells from G1 phase to S phase.

According to our PPI results, we found that *PIMREG* was positively correlated with GTSE1, TPX2, CDC20, CDCA8 and BIRC5, and negatively correlated with EMX2, CRY2, BDH2, TSPAN7 and NR3C2. Coincidentally, most of these genes have been reported in renal cell carcinoma. All the five genes that were positively correlated with *PIMREG* promoted ccRCC. GTSE1 was overexpressed in ccRCC tissues, especially in metastatic samples. In addition, high GTSE1 expression was positively correlated with higher clinical stage, pathological grade and poor prognosis (25). TPX2 is related to the high grade and stage of ccRCC and is an independent predictor of ccRCC recurrence (26). CDC20 is up-regulated in ccRCC with shorter OS and CDCA8 is related to the proliferation and invasion of ccRCC (15, 27). The expression of BIRC5 in renal tubular epithelial cell carcinoma was significantly higher than that in normal renal tissue, which was related to the clinical stage and pathological grade (28). In contrast, the negatively correlated genes all inhibited cancer. BDH2 inhibits the migration and invasion of ccRCC cells by affecting ketone metabolism (29). Patients with high TSPAN7 expression had significantly longer disease free survival (DFS) and tumor specific survival (TSS) (30). Overexpression of NR3C2 inhibits the abilities of proliferation, colony formation, invasion, migration and angiogenesis in ccRCC and reduces the growth of ccRCC xenografts *in vivo* (31). This interesting phenomenon further confirms the tumor promoting effect of *PIMREG* in ccRCC.

CD44 and CD276 are the two immune related genes with the highest correlation with *PIMREG*. CD44 is not only involved in cell proliferation, cell migration, angiogenesis and other normal cell physiological activities, but is closely related to the tumor process (32). Studies have revealed that CD44 is a marker of poor prognosis of ccRCC and is related to sunitinib resistance (33). CD276, also known as PD-1 or PDCD1, plays a key role in inducing and maintaining immune tolerance to organism (34). The majority of tumors can utilize PDCD1 mediated immunosuppression pathway to escape the surveillance of the immune system to promote tumor survival (35). Nivolumab is a programmed death 1 (PD-1) inhibitor, and its combination with ipilimumab (cytotoxic T lymphocyte associated antigen-4 inhibitor) is superior to sunitinib in terms of OS and objective response rates in the treatment of patients with advanced ccRCC (36–39). This suggests that immune checkpoint inhibitor therapy may be the first-line treatment for ccRCC in the future.

It is worth noting that this study still has some limitations. First of all, we only verified it *in vitro*, lacking relevant experimental evidence *in vivo*. Second, we did not explore the molecular mechanism and related signaling pathways by which *PIMREG* regulates the occurrence and development of ccRCC.

## 5 Conclusion

This study proved for the first time that the expression of *PIMREG* was up-regulated in ccRCC and was positively correlated with high TNM stage and poor prognosis. In addition, *PIMREG* can promote the proliferation, migration and invasion of ccRCC cells. *PIMREG* can accelerate the transition from G1 to S stage by regulating cell cycle related proteins, and further promote the occurrence and development of ccRCC. This study explored the promoting effect of *PIMREG* on ccRCC, which is helpful to provide a new therapeutic target for the future treatment.

## Data availability statement

The original contributions presented in the study are included in the article/**Supplementary Material**. Further inquiries can be directed to the corresponding authors.

## Ethics statement

The studies involving human participants were reviewed and approved by the ethics committee of Yantai Yuhuangding Hospital. The patients/participants provided their written informed consent to participate in this study.

## Author contributions

HY, FL, JM, FS, GT, JW, and ZZ: study concept and design. HY, FL, JM, FS, JW, and ZZ: analysis and interpretation of data. HY, FL, JM, GT, JW, and ZZ: preparation of the manuscript. JW and ZZ: critical revision of the manuscript. All authors contributed to the article and approved the submitted version.
