# Supplementary material for: PIMREG is a prognostic biomarker involved in immune microenvironment of clear cell renal cell carcinoma and associated with the transition from G1 phase to S phase
Source: Front Oncol. 2023 Jan 26;13:1035321. doi: 10.3389/fonc.2023.1035321 (PMC9909346; doi:10.3389/fonc.2023.1035321)
Supplement: Supplementary file 1 [file DataSheet_1.zip › supplementary material/gating strategies.docx]

Parameter: FL3-Area-FL3-Area

Gate 1: X: SSC-Height-SSC Y: FSC-Height-FSC

Gate 2: X: FL3-Area-FL3-Area Y: FL3-Height-FL3-Height
