# Supplementary figures and images for: PIMREG is a prognostic biomarker involved in immune microenvironment of clear cell renal cell carcinoma and associated with the transition from G1 phase to S phase
Source: Front Oncol. 2023 Jan 26;13:1035321. doi: 10.3389/fonc.2023.1035321 (PMC9909346; doi:10.3389/fonc.2023.1035321)

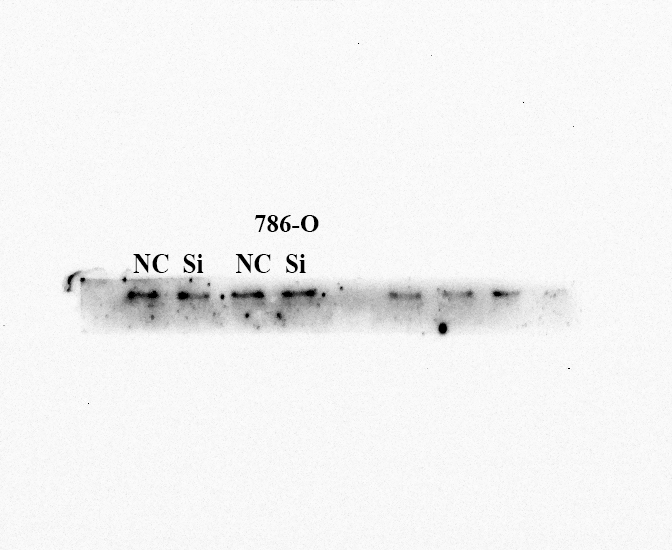

Supplement: Supplementary file 1 [file DataSheet_1.zip › supplementary material/CDK4_220630_172933_08.00.tif]

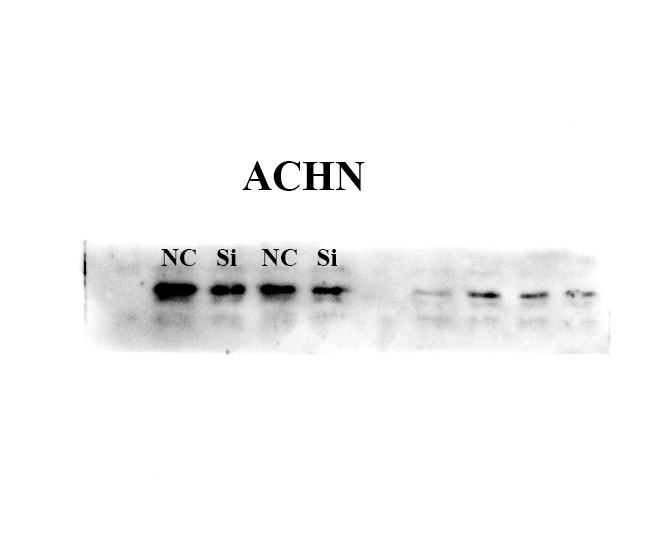

Supplement: Supplementary file 1 [file DataSheet_1.zip › supplementary material/CDK4_220715_172457_02.34.000.tif]

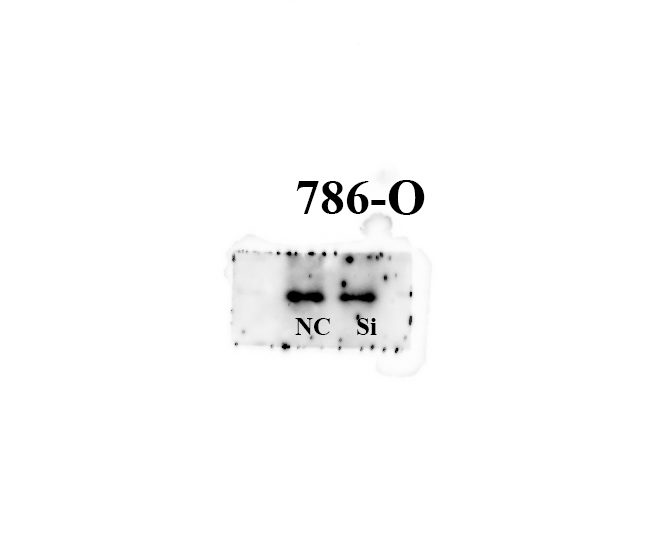

Supplement: Supplementary file 1 [file DataSheet_1.zip › supplementary material/CDK6_220630_175836_08.00.010_1_33103.tif]

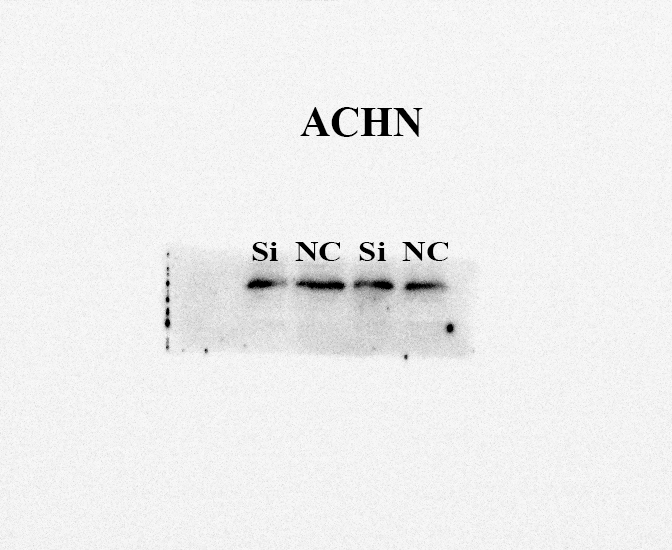

Supplement: Supplementary file 1 [file DataSheet_1.zip › supplementary material/CDK6_220715_173121_00.16.000.tif]

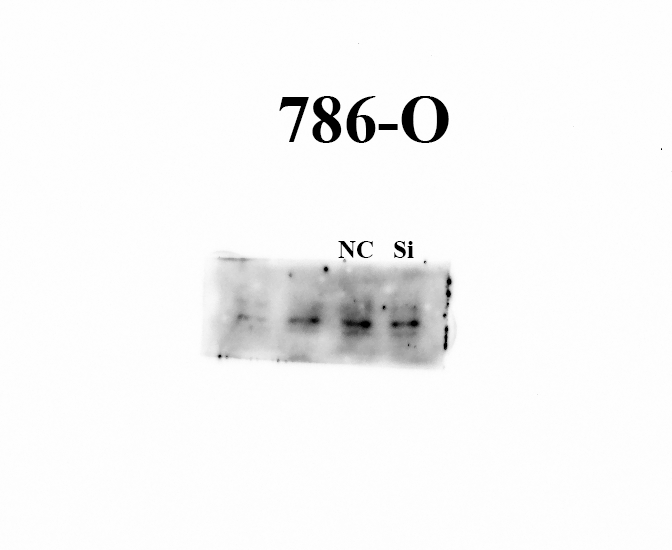

Supplement: Supplementary file 1 [file DataSheet_1.zip › supplementary material/CYCLIND_220630_174448_08.00.010_1_12000.tif]

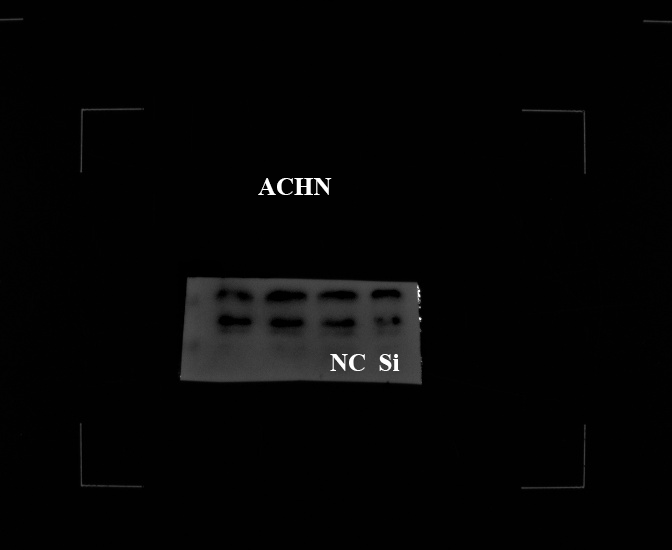

Supplement: Supplementary file 1 [file DataSheet_1.zip › supplementary material/CYCLIND_220715_173439_04.40.000_0_20000.tif]

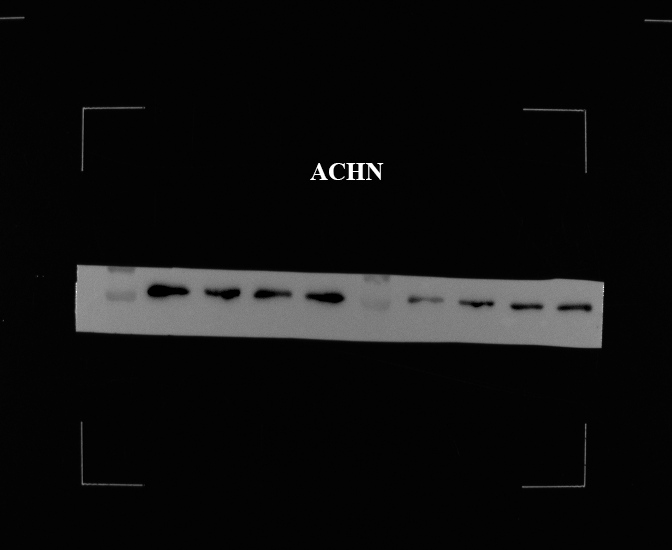

Supplement: Supplementary file 1 [file DataSheet_1.zip › supplementary material/TUBLIN_220715_171353_00.01.000_0_21597.tif]

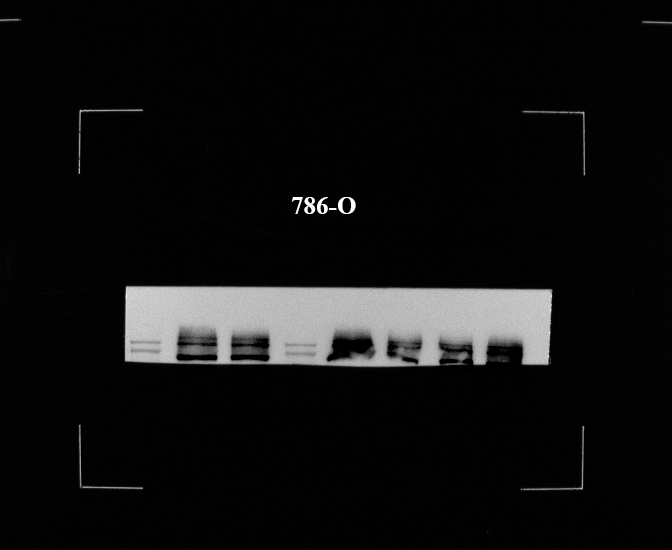

Supplement: Supplementary file 1 [file DataSheet_1.zip › supplementary material/VINCULIN_220630_175609_00.01.000_0_6422.tif]
