# Supplementary material for: PIMREG is a prognostic biomarker involved in immune microenvironment of clear cell renal cell carcinoma and associated with the transition from G1 phase to S phase
Source: Front Oncol. 2023 Jan 26;13:1035321. doi: 10.3389/fonc.2023.1035321 (PMC9909346; doi:10.3389/fonc.2023.1035321)
Supplement: Supplementary file 2 [file Table_1.docx]

| Characteristic | Patients numbers |
| --- | --- |
| T stage | |
| T1 | 13 (40.6%) |
| T2 | 2 (6.3%) |
| T3 | 16 (50%) |
| T4 | 1 (3.1%) |
| N stage | |
| N0 | 30 (93.7%) |
| N1 | 2 (6.3%) |
| M stage | |
| M0 | 32 (100%) |
| M1 | 0 (0%) |
| Clinical Stage | |
| Stage I | 13 (40.6%) |
| Stage II | 2 (6.3%) |
| Stage III | 16 (50%) |
| Stage IV | 1 (3.1%) |
| Gender | |
| Female | 5 (15.6%) |
| Male | 27 (84.4%) |
| Age | |
| <=60 | 19 (59.4%) |
| >60 | 13 (40.6%) |
| Fuhrman Grade | |
| I | 1 (3.1%) |
| II | 16 (50%) |
| III | 13 (40.6%) |
| IV | 0 (0%) |

Supplementary Table.1 Baseline clinical pathological characteristics of the 32 patients.
